# Beating Cardiac Cell Cultures From Different Developmental Stages of Rainbow Trout as a Novel Approach for Replication of Cardiac Fish Viruses

**DOI:** 10.1111/jfd.14080

**Published:** 2025-01-17

**Authors:** Torben Krebs, Julia Bauer, Sarah Graff, Lukas Teich, Markus Sterneberg, Marina Gebert, Henrike Seibel, Bettina Seeger, John Hellmann, Øystein Wessel, Espen Rimstad, Win Surachetpong, Dieter Steinhagen, Verena Jung‐Schroers, Mikolaj Adamek

**Affiliations:** ^1^ Fish Disease Research Unit, Centre for Infection Medicine University of Veterinary Medicine Hannover Hannover Germany; ^2^ Working Group Fish Health and –Welfare, Section Aquaculture and Aquatic Resources Fraunhofer Research Institution for Individualized and Cell‐Based Medical Engineering IMTE Büsum Germany; ^3^ Institute for Food Quality and Food Safety University of Veterinary Medicine Hannover Hannover Germany; ^4^ Environment and Consumer Protection, Fisheries Ecology and Aquaculture North Rhine Westphalian State Agency for Nature Germany; ^5^ Faculty of Veterinary Medicine Norwegian University of Life Sciences Ås Norway; ^6^ Department of Veterinary Microbiology and Immunology, Faculty of Veterinary Medicine Kasetsart University Bangkok Thailand

**Keywords:** cell cultures, heart, PRV‐1, rainbow trout, virus

## Abstract

Piscine orthoreovirus‐1 and 3 (PRV‐1, PRV‐3) cause highly prevalent infection in cultured salmonids and can induce heart and skeletal muscle inflammation (HSMI) resulting in economic losses in aquaculture. However, to date, PRV‐1 and PRV‐3 have withstood replication in continuous cell lines. In this study, we used beating heart cell cultures obtained from different developmental stages of rainbow trout ( *Oncorhynchus mykiss* ) (RTC‐L and RTC‐A) and tested their ability to sustain replication of PRV‐1 and PRV‐3. Furthermore, we compared the replication pattern of the different viruses with those in the newly developed heart fibroblast cell line (RTH‐F) and the traditional established rainbow trout gonad cell line (RTG‐2). Neither RTCs nor RTH‐F cell lines supported replication of PRV‐1 and PRV‐3. Comparative experiments showed varying susceptibility of the novel cultures to viral haemorrhagic septicaemia virus (VHSV), chum salmon reovirus (CSV), infectious pancreatic necrosis virus (IPNV), piscine myocarditis virus (PMCV), salmonid alphavirus 3 (SAV‐3) and tilapia lake virus (TiLV), indicating their usability for work with multiple fish viruses. While confirming the difficulty of replicating PRV‐1 and PRV‐3, the results demonstrate the potential of novel heart‐derived cell cultures as in vitro tools for studying fish viruses.

## 1 Introduction

Viral diseases in farmed aquatic animals are characterised by a high prevalence of infections, which can lead to high mortality rates and the need for specific treatments. These diseases have resulted in significant economic losses and impediments to large‐scale aquaculture globally (Adamek et al. 2019; Dixon et al. 2016; He, Ding, and He 2021). As the aquaculture industry is constantly expanding, the prevalence of disease outbreaks on farms has also increased in recent years (Sun et al. 2024b). Consequently, there are acknowledged gaps in the ability to detect, prevent or treat viral disease outbreaks and the need for more detailed studies of virus replication has become increasingly apparent (Adams 2019; Marsella et al. 2022; Vatne et al. 2024). Viruses represent the second most significant causative agents of diseases leading to farmed fish mortality, with the emergence of highly virulent viruses in the salmonid industry increasing over the past decade (Kibenge 2019).

While some viruses that infect a wide range of tissues, such as birnaviruses and rhabdoviruses, have been successfully isolated and reisolated in various cell cultures (Choi et al. 2024; Dopazo 2020; Panzarin et al. 2018; Wolf, Dunbar, and Snieszko 1960), the isolation and replication of other viruses of salmonids has shown to be increasingly challenging. This has led to a growing need for new tools for virus isolation, particularly the development of cell cultures from specific cell types to isolate and propagate different viruses (Noguera et al. 2021; Pan et al. 2009). Viruses have evolved together with their hosts, with specific host‐pathogen interactions and affinity for infection of particular cell types or cells in specific stages of the cell cycle. Consequently, the more complex infection mechanisms that lead to successful viral replication of certain viruses make isolation difficult (Dhamotharan et al. 2020; Schönherz, Lorenzen, and Einer‐Jensen 2013). An illustrative case is that of the cardiac virus piscine orthoreovirus‐1 (PRV‐1) and 3 (PRV‐3), where the disease manifests in a gradual, stepwise manner (Ivanova et al. 2024; Sørensen et al. 2023; Vatne et al. 2024) and which have never been successfully cultured in vitro beyond several passages in erythrocytes (Pham et al. 2020; Tsoulia et al. 2024).

The piscine orthoreoviruses are classified in the order *Reovirales*, family *Spinareoviridae* and genus *Orthoreovirus* (Vallejos‐Vidal et al. 2022). Phylogenetic analyses and host species specificity demonstrated that PRV can be divided into three different genotypes: PRV‐1, PRV‐2 and PRV‐3. PRV is regarded as an emerging virus that causes heart and skeletal muscle inflammation (HSMI) in Atlantic salmon ( *Salmo salar* ) (PRV‐1) (Wessel et al. 2017), erythrocytic inclusion body syndrome (EIBS) in Japanese coho salmon ( *Oncorhynchus kisutch* ) (PRV‐2) (Takano et al. 2016) and HSMI‐like infections in rainbow trout (PRV‐3) (Olsen et al. 2015). The disease HSMI in Atlantic salmon is characterised by different types of carditis, myositis, myocardial necrosis and necrosis of the red skeleton muscle (Kongtorp, Taksdal, and Lyngøy 2004). It is assumed that PRVs replicate in red blood cells and subsequently infect cardiomyocytes (Finstad et al. 2014, 2012). The initial description of PRV‐1 was published in 2010 (Palacios et al. 2010), but the disease was first detected in Norway in 1999 (Kongtorp, Taksdal, and Lyngøy 2004). PRV‐1 is a non‐enveloped, spherical virus with a ten‐segmented, double‐stranded RNA genome packaged in a double‐layered, icosahedral protein capsid (Markussen et al. 2013; Wessel et al. 2017). In Atlantic salmon populations, the disease typically results in moderate mortality, reaching up to 20% in severe cases (Kongtorp, Taksdal, and Lyngøy 2004). To prevent PRV‐1 outbreaks, numerous studies are currently looking into the development of vaccines against PRV‐1, but no vaccines are yet commercially available. The tools for early detection and replication of PRV‐1 and other health threats are still in their infancy (Adamek et al. 2019; Ivanova et al. 2024; Malik et al. 2021).

The present study assesses the efficacy of a recently developed myocardial cell culture by Krebs et al. (2025) and Graff et al. (in preparation) derived from rainbow trout larval hearts (RTC‐L) and one‐year‐old rainbow trout hearts (RTC‐A), as well as a permanent non‐myocardial (fibroblastic) cell line (RTH‐F) obtained from rainbow trout heart, as an in vitro tool for PRV‐1 and other fish viruses' replication. Cell cultures were infected with chum salmon reovirus (CSV), infectious pancreatic necrosis virus (IPNV), piscine myocarditis virus (PMCV), salmonid alphavirus 3 (SAV‐3), tilapia lake virus (TiLV) and viral haemorrhagic septicaemia virus (VHSV), with variations in temperature and incubation time. Viral load and gene expression analysis for selected antiviral genes were performed. Specifically, the performance of the newly developed heart cell cultures was compared with the established RTG‐2 cell line in infection experiments.

## 2 Materials and Methods

### 2.1 Fish and Larvae Husbandry

In the present study, the experiments were conducted using non‐feeding, one‐week post‐hatching rainbow trout larvae in the yolk‐sac stage and one‐year‐old rainbow trout. The rainbow trout eggs and adult trout were sourced from multiple commercial fish farms in Germany. Eggs were obtained in the eyed stage and hatched at the Fish Disease Research Unit, University of Veterinary Medicine Hannover. The larvae and older rainbow trout were maintained in a flow‐through aquaculture system at 10°C–12°C with suitable aeration. The research was conducted on non‐feeding larvae and tissues collected from fish post‐mortem in accordance with local and international animal experimentation legislation (Article 4, TSchG Killing for scientific purposes, Annunciation number: TiHo‐T‐2022‐3).

### 2.2 Cell Cultures

Beating cell cultures were prepared from rainbow trout hearts (RTC) using a modified protocol for isolating primary cardiomyocytes from zebrafish hearts (Sander et al. 2013) as published recently by Krebs et al. (2025). Briefly: A total of 210 hearts from yolk sac stage larvae exhibiting healthy appearance and non‐feeding behaviour were used for each 24‐well plate (RTC‐L). To obtain cell cultures derived from the hearts of one‐year‐old rainbow trout, 15 hearts were utilised for each 24‐well plate (RTC‐A). Prior to extraction of the hearts rainbow trout larvae and one‐year‐old rainbow trout were anaesthetised using an overdose (0.5 g/L) of Tricaine (PHARMAQ). As described in the modified protocol of Krebs et al. (2025), after several washing steps and a digestion process, cardiomyocytes were plated into all wells of a 24‐well plate containing 500 μL of advanced DMEM/F‐12 plating medium (Thermo Fisher Scientific) and incubated at 15°C and 2% CO₂. On a weekly basis, 300 μL of the medium was removed and replaced with an equal volume of freshly prepared medium. Following a period of two to four weeks, evidence of cell contraction in each well was observed, indicating that the cultures were ready to be used in the planned experiments. In addition, cell cultures of the well‐characterised cell line rainbow trout gonad (RTG‐2) and newly established cell line rainbow trout heart (RTH‐F grown from heart explants of juvenile rainbow trout and passaged over 100 times) from the Fish Disease Research Unit of the University of Veterinary Medicine Hannover, Germany, which is described for the first time in this paper, were used for the planned experiments. RTG‐2 and RTH‐F cells were plated into 24‐well plates, grown for 24 h and utilised upon reaching 95% confluence before infection. The cells were cultured in Minimum Essential Medium Eagle (MEM) (Sigma) medium with 10% FCS, 10 IU/mL penicillin and 100 mg/mL streptomycin. Cultures were incubated at 15°C and 2% CO_2_.

### 2.3 Infection of Rainbow Trout Larval Cardiac Cell Cultures With PRV‐1 and PRV‐3

Three‐week‐old contracting rainbow trout larval cardiac cell cultures (RTC‐L) were infected with PRV‐1 (Norway) and PRV‐3 (Germany) isolates at a dilution factor of 1:20. 10 μL of the viral suspension was added to each well containing 490 μL advanced DMEM/F‐12 plating medium (Thermo Fisher Scientific), thereby infecting the cardiomyocytes. 10 μL of cell culture medium was added in control wells. Cell cultures were incubated at 15°C and 2% CO_2_ during the infection experiment. Sampling was conducted approximately 30 min following infection (0 days post‐infection (dpi)), at 3 dpi, and on a weekly basis at 7, 14 and 21 dpi. For sampling the medium, 100 μL of the medium was collected and transferred into 1 mL TRI reagent (Sigma) for further analysis. For sampling the cells, the medium was aspirated using a pipette and then 1 mL of 50 FU ml^−1^ nattokinase (Aportha) dissolved in PBS (Sigma) was added to the wells. After 10 min, a free‐floating cell layer was observed and the entire volume was transferred to a 2 mL reaction tube and subjected to a 5‐min centrifugation at low speed (300 x g, 4°C). Following centrifugation, the supernatant was removed, and the cell pellet was resuspended in 1 mL TRI reagent (Sigma). The samples were stored in a freezer at minus 80°C for subsequent analysis.

### 2.4 Infection of Cardiac Cell Cultures From One‐Year‐Old Rainbow Trout With PRV‐1 and VHSV

Two‐ to three‐week‐old contracting cell cultures from one‐year‐old rainbow trout (RTC‐A) were infected with PRV‐1 (Norway) and VHSV (F13, Germany) isolates. To adjust the virus concentration to a similar concentration as in the larval culture experiment, a dilution factor of 1:200 was used. Following the infection, the cells were maintained at 8°C. Three days prior to the introduction of the respective virus, the advanced DMEM/F‐12 plating medium (Thermo Fisher Scientific) was replaced with an L‐15 plating medium (Sigma) containing the same supplements as the previous cell culture medium. This allowed the cells to adapt to the new medium. A preliminary trial was conducted prior to the infection experiment, in which the change from advanced DMEM/F‐12 (Thermo Fisher Scientific) to L‐15 plating medium (Sigma) was tested on other contracting cell cultures of rainbow trout of the same age. The results demonstrated that there was no observable change in vitality or contraction activity. For the infection experiment, first 400 μL of fresh L‐15 medium (Sigma) and then 100 μL of virus suspension were added to each well. In the control group, 100 μL of fresh medium was added instead of the virus suspension. Sampling was conducted approximately 30 min following infection (0 dpi), as well as on day 3 dpi and then weekly until day 21 dpi for the VHSV experiment and until day 28 dpi for the PRV‐1 experiment. Sampling of cells and medium was performed as detailed above. All samples were then frozen at minus 80°C for subsequent analyses.

### 2.5 Comparison of Different Rainbow Trout Cell Cultures After Infection With Various Viruses

Three cell lines of rainbow trout, namely RTG‐2, RTH‐F and RTC‐A, were selected for comparison and infected with various viruses. The used virus isolates in a dilution factor of 1:20 were chum salmon reovirus (CSV, isolate 017.94, USA), infectious pancreatic necrosis virus (IPNV, serotype Sp, Germany), piscine myocarditis virus (PMCV, Norway), salmonid alphavirus 3 (SAV‐3, Norway), tilapia lake virus (TiLV isolate VETKU‐TV01, Thailand) and viral haemorrhagic septicaemia virus (VHSV, F13, Germany). All cell lines were grown to full confluence in all wells and special care was taken to ensure that contractions were visible in the RTC cells. This was the case in all of them after three weeks. The RTG‐2 and RTH‐F cell lines were maintained in a MEM plating medium (Sigma), whereas the RTC cell cultures were maintained in an advanced DMEM/F‐12 plating medium (Thermo Fisher Scientific). The cell cultures infected with CSV, IPNV and VHSV were adapted to an L‐15 plating medium (Sigma) as detailed above and maintained at 8°C. Advanced DMEM/F‐12 plating medium (Thermo Fisher Scientific) was used for infection experiments with PMCV, SAV‐3 and TiLV at 15°C. The cells were infected with 100 μL of virus suspension, which was added to either fresh L‐15 medium (Sigma) or advanced DMEM/F‐12 plating medium (Thermo Fisher Scientific) in each well. The control group received 100 μL of the plating medium instead of the virus suspension. Samples were taken approximately 30 min after infection (0 dpi) and at 1 dpi and 5 dpi. Only the cell samples were obtained. For RTG‐2 and RTH‐F, the cell medium was removed and the cells were lysed with 1 mL of TRI reagent (Sigma). Sampling of cells from RTC‐A was performed as detailed above. All samples were frozen at minus 80°C for further analyses.

### 2.6 Molecular Biology Analysis

For quantitative PCR analysis, RNA was isolated from the cell culture samples using TRI‐Reagent (Sigma) in accordance with the manufacturer's instructions. cDNA was transcribed using the Maxima First Strand cDNA Synthesis Kit (Thermo Fisher Scientific) and diluted 1:20 with nuclease‐free water prior to quantitative PCR.

Quantitative PCR was performed in duplicates using the Maxima Probe or Maxima SYBR Green 2x Mastermix (Thermo Fisher Scientific) on a StepOne Thermocycler (Applied Biosystems). The reaction mix contained 1x the Maxima Probe or Maxima SYBR Green Mastermix (containing 10 nM ROX), 0.2 μM of each primer and 3.0 μL cDNA (1:20 dilution). Nuclease‐free water was added to a final volume of 10 μL. For the SYBR Green runs, the amplification programme consisted of an initial denaturation phase of 10 min at 95°C, 40 identical cycles of denaturation for 30 s at 95°C, annealing at 55°C for 30 s and a final elongation at 72°C for 30 s. A melting curve was performed at the end of each SYBR Green run. The amplification programme for the Probe runs included an initial denaturation at 95°C for 10 min, followed by 40 cycles of denaturation at 95°C for 30 s and annealing/elongation at 56°C for 30 s. For quantification, recombinant DNA plasmid standard curves were prepared in the range of 10 to 10^7^ gene copies and subsequently employed for the quantification of the copy number in each sample. Measurements were normalised by the determination of two reference genes (elongation factor 1α and 39S ribosomal protein L40). For quantification, copy numbers of gene‐specific RNA were normalised against 10^4^ copies of the reference gene rainbow trout elongation factor 1α (*ef1α*) and 10^3^ copies of the reference gene Atlantic salmon 39S ribosomal protein L40 (*39SL40*). The targeted genes were selected based on the existing literature. In these studies, the genes were observed to be regulated in response to viral infection or antiviral activity. The present study concentrated on the proinflammatory genes *ifng2*, *il‐6 L*, antiviral genes *isg15*, *mx1*, *vig1* and cellular stress marker *sacs*. Primer sequences of the selected genes are shown in Supplementary Table 1.

### 2.7 Statistics

Statistical analyses were conducted using Systat software SigmaPlot 12. Log10 data were tested for normal distribution and equality of variances. Viral load data (Log10) were tested with a one‐way analysis of variance (ANOVA). Significant differences (*p* < 0.05) between 0 dpi and different time points are pointed out by a *. For gene expression analysis significant differences (p < 0.05) between control and infected cells (*) were assessed using a two‐way ANOVA, followed by pairwise multiple comparisons using the Holm–Sidak method if the data showed a normal distribution. In the event of a non‐normal distribution of the data, a Kruskal–Wallis one‐way ANOVA on ranks was employed instead.

## 3 Results

### 3.1 No PRV‐1 and PRV‐3 Replication in Rainbow Trout Larvae Cells (RTC‐L)

**FIGURE 1:**
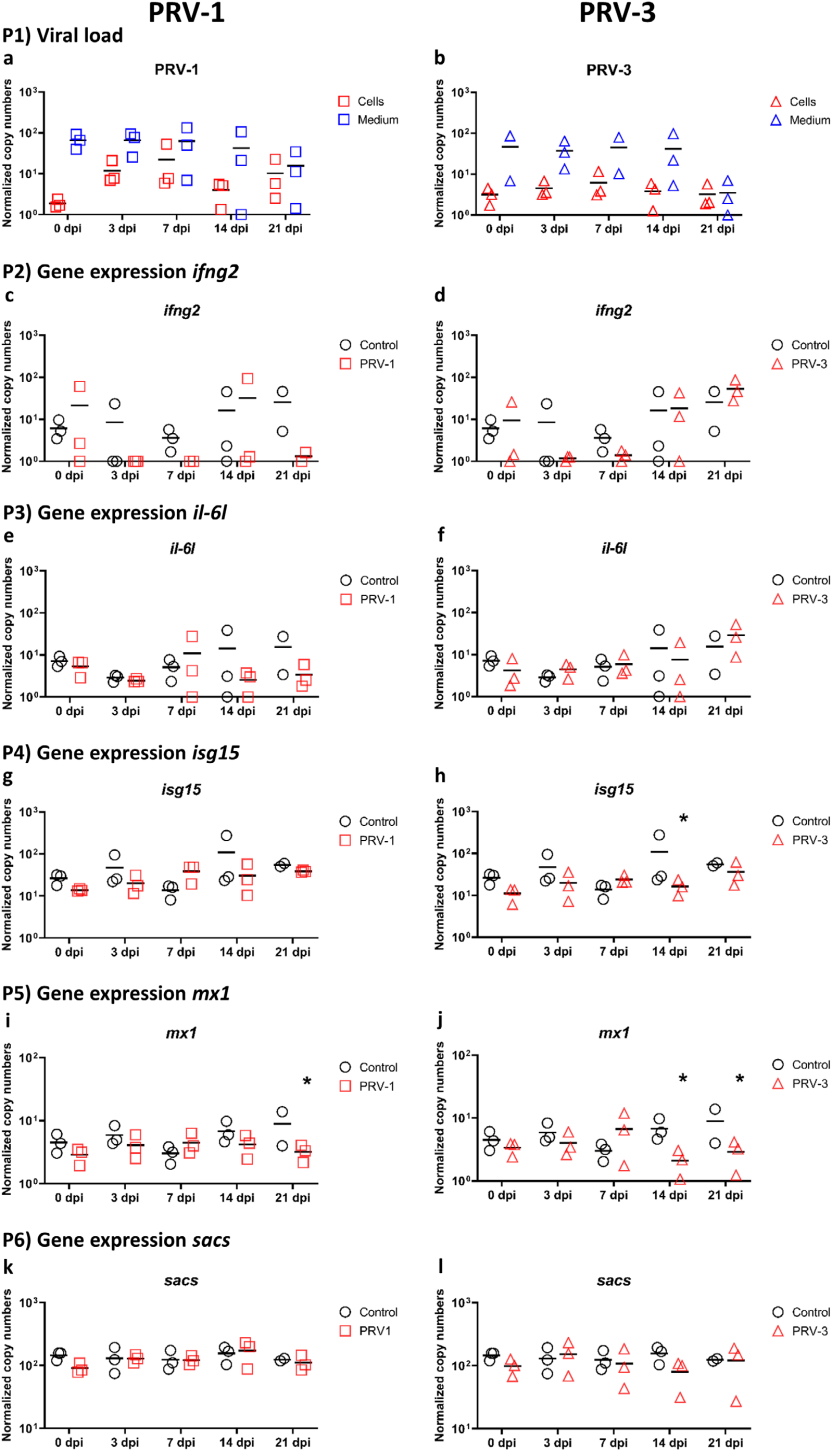
Levels of RNA encoding for viral RNA (Panel P1a: PRV‐1, Panel P1b: PRV‐3) and rainbow trout genes related to antiviral response (Panel P2c‐d: *Ifng2*, Panel P3e‐f: *Il‐6 L*, Panel P4g‐h: *Isg15*, Panel P5i‐j: *Mx1*, Panel P6k‐l: *Sacs*) after 0, 3, 7, 14 and 21 days post‐infection (dpi) of three weeks old contracting rainbow trout larval cardiomyocytes cultures (RTC‐L) infected with either PRV‐1 or PRV‐3 in 15°C. Results are presented as scatter plots with all data points and the mean shown. Data are presented as normalised copy numbers. * indicates statistically significant differences in expression levels of either infected cell cultures compared to 0 dpi (Panel P1) or infected cell cultures compared to uninfected controls (Panel P2‐6) at *p* < 0.05. Statistical analysis for viral data (Panel P1) was performed using one‐way ANOVA. Where data were not normally distributed, Kruskal–Wallis one‐way ANOVA on ranks was used. For gene expression data (Panel P2‐6) statistical analysis was performed using two‐way ANOVA with multiple comparison tests using the Holm–Sidak method. Missing data points (not shown in plots) were corrected for statistical tests using SigmaPlot software.

No cytopathic effect (CPE) or viral replication was detected in contracting RTC‐L cultures infected with PRV‐1 or PRV‐3 at 15°C. For PRV‐1, both in the medium and in the cells, the average viral RNA copy load was a maximum of 10^2^, but in the cells, the numbers were slightly lower (Figure 1 P1a). A similar outcome was observed for PRV‐3 (Figure 1 P1b). The proinflammatory genes *ifng2* (Figure 1 P2c‐d), *il‐6 L* (Figure 1 P3e‐f), antiviral genes *isg15* (Figure 1 P4g‐h), *mx1* (Figure 1 P5i‐j) and cellular stress marker *sacs* (Figure 1 P6k‐l) were analysed at gene expression level in cells inoculated with PRV‐1 and PRV‐3. Infection with PRV‐1 and PRV‐3 showed no clear differences in gene expression between control and infected cell culture, except for significant differences in the *mx1* gene for PRV‐1 at 21 dpi (Figure 1 P5i) and at 14 and 21 dpi for PRV‐3 (Figure 1 P5j). Furthermore, a significant difference can be observed between control and infected cells for *isg15* in the context of PRV‐3 infection (Figure 1 P4h).

### 3.2 VHSV Replication in Rainbow Trout Cells (RTC‐A) at 8°C, While PRV‐1 Showed No Replication

**FIGURE 2:**
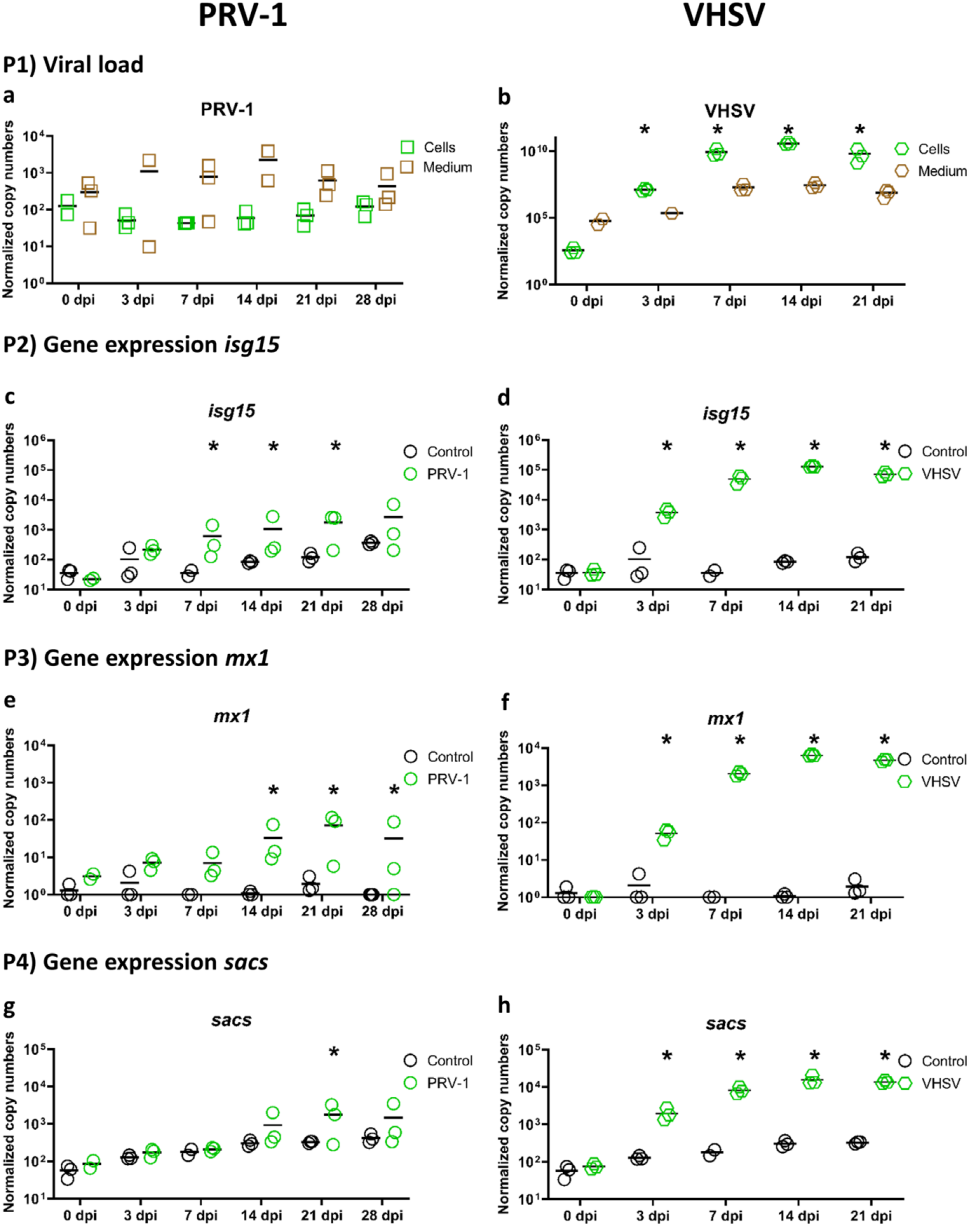
Levels of RNA encoding for viral RNA (Panel P1a: PRV‐1, Panel P1b: VHSV) and rainbow trout genes related to antiviral response (Panel P2c‐d: *Isg15*, Panel P3e‐f: *Mx1*, Panel P4g‐h: *Sacs*) after 0, 3, 7, 14, 21 and 28 days post‐infection (dpi) of four weeks old contracting rainbow trout cardiomyocytes cultures (RTC‐A) infected with either PRV‐1 or VHSV in 8°C. Results are presented as scatter plots with all data points and the mean shown. Data are presented as normalised copy numbers. * indicates statistically significant differences in expression levels of either infected cell cultures compared to 0 dpi (Panel P1) or infected cell cultures compared to uninfected controls (Panel P2‐4) at *p* < 0.05. Statistical analysis for viral data (Panel P1) was performed using one‐way ANOVA. Where data were not normally distributed, Kruskal–Wallis one‐way ANOVA on ranks was used. For gene expression data (Panel P2‐4) statistical analysis was performed using two‐way ANOVA with multiple comparison tests using the Holm–Sidak method. Missing data points (not shown in plots) were corrected for statistical tests using SigmaPlot software.

Contracting RTC‐A cultures were infected at 8°C with PRV‐1 or VHSV and incubated 28 or 21 dpi, respectively. In this experiment, following the results of the infection experiment with PRV‐1 and PRV‐3 at 15°C, we investigated whether PRV‐1 could replicate at lower temperatures in our heart cell cultures. VHSV was used as a positive control to see if a fish virus known to be able to replicate in various cell lines could replicate in the cell cultures. Both viral load and gene expression results demonstrated that VHSV replicated in contracting heart cell cultures of rainbow trout infected at 8°C, but no CPE was observed. In the cells, the viral gene copy load was already 10^7^ after three dpi and increased to 10^10^ over the course of the experiment. The viral gene copy load in the cell culture medium ranged between 10^4^ and 10^8^ (Figure 2, P1b). Analysis of the gene expression level of *isg15* (Figure 2 P2d), *mx1* (Figure 2 P3f) and *sacs* (Figure 2 P4h) showed upregulation following VHSV infection. The three genes exhibited a similar pattern of expression. From 3 dpi onwards, the infected cells were statistically significantly different from the control. The mean of normalised copy numbers in the controls were 10 for *mx1* and 10^2^ for *isg15* and *sacs*. Infection with VHSV resulted in a notable increase in the copy number values, reaching 10^5^ for *isg15*, 10^4^ for *sacs*, and nearly 10^4^ for *mx1*. Therefore, the gene expression profile aligned with the viral load dynamics. The results of the PRV‐1 infection at 8°C indicated that there was no CPE and viral replication in the cells. The mean values remain at approximately 10^2^ throughout the 28‐day period post‐infection. Furthermore, no significant increase in PRV‐1 could be observed in the cell medium (Figure 2, P1a). At the gene expression level, a slight upregulation of *isg15* (Figure 2 P2c), *mx1* (Figure 2 P3e) and *sacs* (Figure 2 P4g) was observed. Statistically significant differences were identified, although the observed increase in expression was relatively modest in comparison to that observed in VHSV infection and the scatter of data points was relatively large.

### 3.3 Comparative Analysis of Viral Replication and Gene Expression in RTC‐A, RTG‐2 and RTH‐F Cell Lines

**FIGURE 3:**
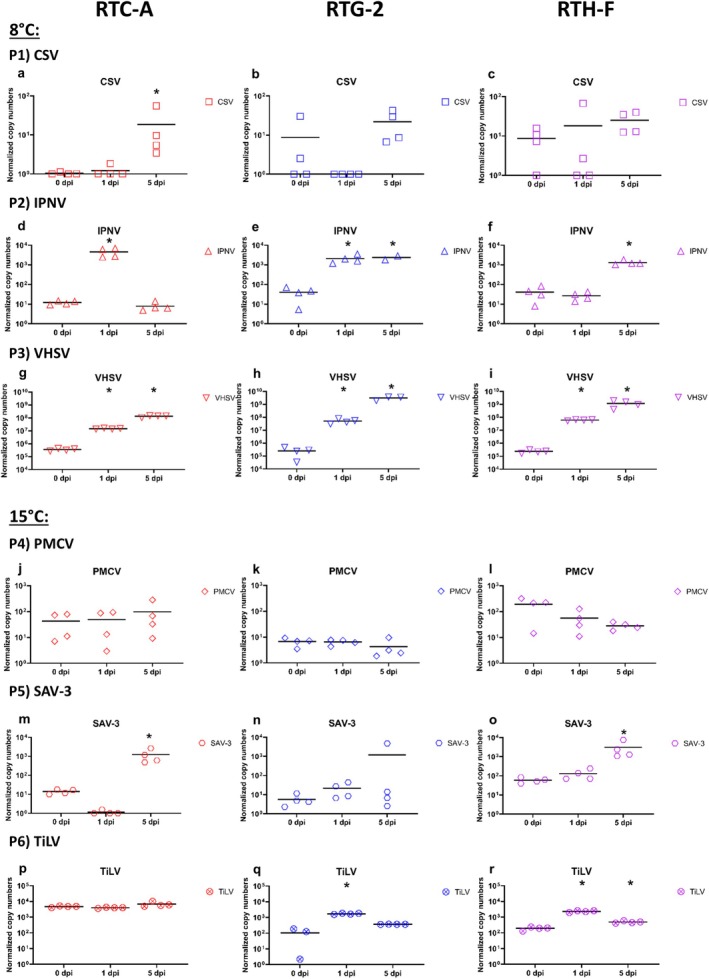
Levels of viral RNA encoding for different viruses CSV (Panel P1a‐c), IPNV (Panel P2d‐f), VHSV (Panel P3g‐i), PMCV (Panel P4j‐l), SAV‐3 (Panel P5m‐o) and TiLV (Panel P6p‐r) after infection of three different rainbow trout cell cultures (rainbow trout cardiomyocyte cell culture (RTC‐A), rainbow trout gonad cell culture (RTG‐2) and rainbow trout heart cell culture (RTH‐F)) over a period of five days (5 dpi). Results are presented as scatter plots with all data points and the mean shown. Data are presented as normalised copy numbers. * indicates statistically significant differences in expression levels of infected cell cultures compared to 0 dpi at *p* < 0.05. Statistical analysis was performed using one‐way ANOVA. Where data were not normally distributed, Kruskal–Wallis one‐way ANOVA on ranks was used. Missing data points (not shown in plots) were corrected for statistical tests using SigmaPlot software.

**FIGURE 4:**
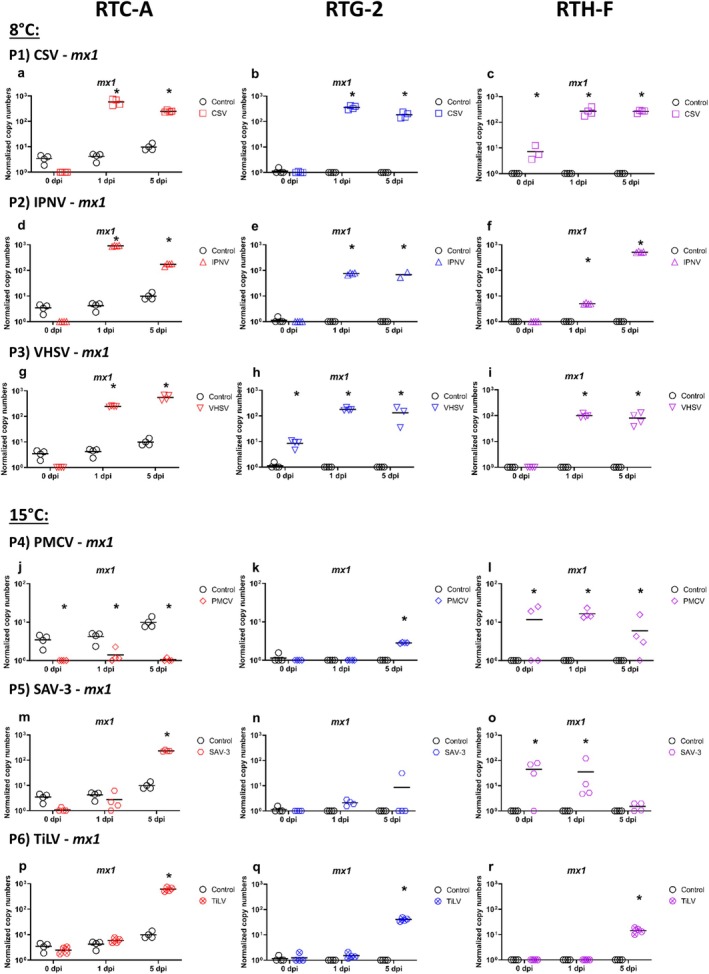
Levels of RNA encoding for *mx1* gene related to antiviral response after infection of three different rainbow trout cell cultures (rainbow trout cardiomyocyte cell culture (RTC‐A), rainbow trout gonad cell culture (RTG‐2) and rainbow trout heart cell culture (RTH‐F)) with different viruses CSV (Panel P1a‐c), IPNV (Panel P2d‐f), VHSV (Panel P3g‐i), PMCV (Panel P4j‐l), SAV‐3 (Panel P5m‐o) and TiLV (Panel P6p‐r) over a period of five days (5 dpi). Results are presented as scatter plots with all data points and the mean shown. Data are presented as normalised copy numbers. * indicates statistically significant differences in expression levels of infected cell cultures compared to uninfected controls at p < 0.05. Statistical analysis was performed using two‐way ANOVA with multiple comparison tests using the Holm–Sidak method. Missing data points (not shown in plots) were corrected for statistical tests using SigmaPlot software.

**FIGURE 5:**
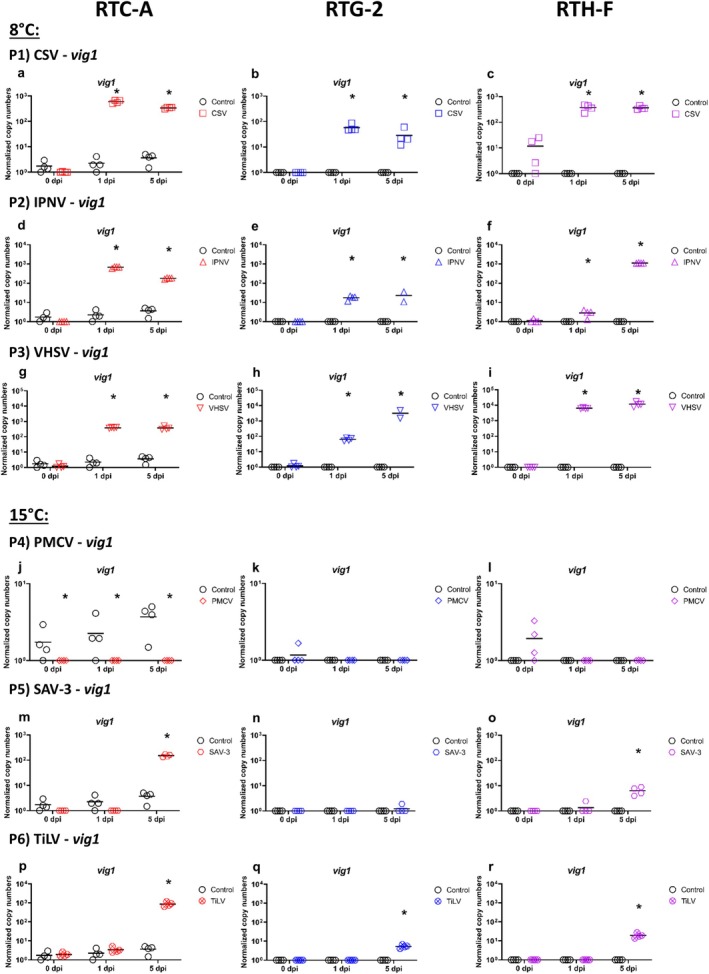
Levels of RNA encoding for *vig1* gene related to antiviral response after infection of three different rainbow trout cell cultures (rainbow trout cardiomyocyte cell culture (RTC‐A), rainbow trout gonad cell culture (RTG‐2) and rainbow trout heart cell culture (RTH‐F)) with different viruses CSV (Panel P1a‐c), IPNV (Panel P2d‐f), VHSV (Panel P3g‐i), PMCV (Panel P4j‐l), SAV‐3 (Panel P5m‐o) and TiLV (Panel P6p‐r) over a period of five days (5 dpi). Results are presented as scatter plots with all data points and the mean shown. Data are presented as normalised copy numbers. * indicates statistically significant differences in expression levels of infected cell cultures compared to uninfected controls at p < 0.05. Statistical analysis was performed using two‐way ANOVA with multiple comparison tests using the Holm–Sidak method. Missing data points (not shown in plots) were corrected for statistical tests using SigmaPlot software.

A comparison of the three cell lines RTC‐A, RTG‐2 and RTH‐F infected with CSV, IPNV and VHSV at 8°C yielded contrasting results. CPE was observed for IPNV and VHSV but not for CSV in RTG‐2 and RTH‐F. For RTC‐A, it was difficult to obtain clear indications of CPE as the cultures were grown on a fibrin gel coating which impairs the CPE observation (Table 1). For CSV, there was no evidence of viral replication in RTH‐F (Figure 3 P1c). In contrast, a slight increase in viral load was observed in RTC‐A (Figure 3 P1a) and RTG‐2 (Figure 3 P1b). Despite this, all three cell lines show upregulation of both, *mx1* (Figure 4 P1a‐c) and *vig1* (Figure 5 P1a‐c), at 1 dpi and 5 dpi at comparable levels of 10^2^ to10^3^ normalised copy numbers. Regarding IPNV (Figure 3 P2d‐f), the course of the experiment revealed disparate viral loads in the three cell cultures. In the RTC‐A cell culture (Figure 3 P2d), the viral load was observed to be at its highest at 1 dpi, exhibiting a significant difference from the initial level at 0 dpi, and subsequently returning to the initial level at 5 dpi. Compared to RTC‐A, the viral load of IPNV increased in RTG‐2, reaching normalised copy numbers between 10^3^ and 10^4^ at 1 dpi (Figure 3 P2e), and was stable at the same level at 5 dpi. In RTH‐F (Figure 3 P2f), viral load increased most at 5 dpi. The gene expression of *mx1* (Figure 4 P2d‐f) and *vig1* (Figure 5 P2d‐f) in response to IPNV infection exhibits a pattern that correlates with the viral load. However, for RTC‐A, *mx1* (Figure 4 P2d) and *vig1* (Figure 5 P2d) demonstrate still higher expression at 5 dpi, diverging from the observed trend in IPNV viral load. The most pronounced replication of the virus was evident in all cell lines when using VHSV (Figure 3 P3g‐i). The viral load increased significantly and reached the highest viral load ranging between 10^9^ and 10^10^ at 5 dpi. The expression of *mx1* (Figure 4 P3g‐i) and *vig1* (Figure 5 P3g‐i) exhibited a consistent pattern across all cell lines, with the genes already showing clear upregulation at 1 dpi and maintaining this expression level up to 5 dpi. An overview of the main results of the infection experiment is given in Table 1.

**TABLE 1:** Summary table of the key findings of the infection experiment of the three cell lines RTC‐A, RTG‐2 and RTH‐F with different viruses. **+** indicates the occurrence of CPE, an increase in viral load or increased expression of *mx1* or *vig1*. **‐** shows no CPE or no change in viral load or gene expression. n.a—not applicable, cultures were grown on fibrin gel coating which impairs the CPE observation.

	Cytopathic effect			Increase of viral load			Increase of *mx1* expression			Increase of *vig1* expression		
	RTC‐A	RTG‐2	RTH‐F	RTC‐A	RTG‐2	RTH‐F	RTC‐A	RTG‐2	RTH‐F	RTC‐A	RTG‐2	RTH‐F
CSV	n.a	−	−	+	+	−	+	+	+	+	+	+
IPNV	n.a	+	+	+	+	+	+	+	+	+	+	+
VHSV	n.a	+	+	+	+	+	+	+	+	+	+	+
PMCV	n.a	−	−	−	−	−	−	+	+	−	−	−
SAV‐3	n.a	−	−	+	−	+	+	−	+	+	−	+
TiLV	n.a	−	−	−	+	+	+	+	+	+	+	+

The infections of the cell cultures RTC‐A and cell lines RTG‐2 and RTH‐F with PMCV, SAV‐3 and TiLV at 15°C also yielded contrasting results, with no CPE observed in any of the three cultures. No increase in viral load was observed in all cell cultures infected with PMCV (Figure 3 P4j‐l). Although the gene expression results of *mx1* (Figure 4 P4j‐l) and *vig1* (Figure 5 P4j‐l) showed fluctuations, the results do not indicate a replication of PMCV but underlie the scatter of values. In the case of RTC‐A, the values for *mx1* (Figure 4 P4j) and *vig1* (Figure 5 P4j) are even lower than the controls, but in the same range of 1 to 10. (Figure 4 P4j‐l). For SAV‐3 (Figure 3 P5m‐o), the viral load increased over the course of the experiment in RTC‐A (Figure 3 P5m) and RTH‐F (Figure 3 P5o). In contrast, the results for RTG‐2 (Figure 3 P5n) were variable. One sample demonstrated a markedly increased viral load, while the remaining samples at 5 dpi exhibited no discernible increase in viral load. Furthermore, the expression of *mx1* (Figure 4 P5n) exhibited a similar pattern to that of the viral load, whereas *vig1* (Figure 5 P5n) in RTG‐2 did not provide any evidence of viral replication. In RTC‐A, both *mx1* (Figure 4 P5m) and *vig1* (Figure 5 P5m) exhibited upregulation at 5 dpi. Interestingly, in RTH‐F, the expression of *mx1* and *vig1* showed contrasting patterns. M*x1* displayed a tendency towards downregulation throughout the course of infection (Figure 4 P5o), whereas *vig1* demonstrated an increase in expression (Figure 5 P5o). An increase in viral TiLV load was not observed in RTC‐A (Figure 3 P6p). In contrast, a slight increase in viral load was observed in RTG‐2 (Figure 3 P6q) and RTH‐F (Figure 3 P6r). At the gene expression level, *mx1* (Figure 4 P6p‐r) and *vig1* (Figure 5 P6p‐r) exhibited comparable upregulation in all cell lines, reaching a peak at 5 dpi. The key findings obtained from this section of the infection experiment are summarised in Table 1.

## 4 Discussion

In the present study, the potential of PRV replication was investigated in beating heart cell cultures derived from rainbow trout at different stages of development, using different temperatures and compared to the replication of other fish viruses. Comparative analyses were also carried out between heart cell cultures and established rainbow trout cell lines regarding virus replication. We chose to use rainbow trout heart cell cultures for our studies because both natural infections and experimental studies have shown that PRV mainly infects species of the genus *Oncorhynchus* and *Salmo* within the salmonid family (Polinski et al. 2020). For example, in the studies by Kibenge et al. (2013), Cartagena et al. (2018) and Purcell et al. (2018), PRV‐1 genetic material was detected in rainbow trout, confirming that rainbow trout can be a host for PRV‐1 and be a potential suitable for our experiments. The fact that rainbow trout is one of the main hosts for PRV‐3 infection also verified our decision to use heart cell cultures from rainbow trout (Adamek et al. 2019; Cartagena et al. 2018; Dhamotharan et al. 2018). Furthermore, during the project, it became clear that beating heart cell cultures from rainbow trout were the most reliable in the laboratory. In addition to erythrocytes as the primary target, cardiomyocytes are also target cells for PRV in salmonids, as confirmed by the presence of PRV antigens in these cells (Finstad et al. 2014, 2012). Nevertheless, our studies demonstrated that no replication occurred in vitro in the cardiomyocyte cultures of rainbow trout larvae. Neither PRV‐1 nor PRV‐3 were able to replicate in beating heart cell cultures from rainbow trout larvae (RTC‐L) at 15°C. This was concluded from the absence of any indicators of viral replication, that is neither the viral load in the cells or in the cell medium nor the expression of the antiviral genes analysed showed any significant changes during the infection experiment. Combined with the same negative results obtained with Atlantic salmon ( *Salmo salar* ) and brown trout ( *Salmo trutta* ) cultures (data not shown), our hypothesis that the development of a cell culture of the heart (beating cardiomyocyte culture) would provide a new tool to study PRV replication in vitro was refuted. Previous research has demonstrated that salmonids are susceptible to PRV‐1 during the early stages of development and can also transmit the virus. However, it has also been demonstrated that the susceptibility varies between the respective salmonid species (Kannimuthu et al. 2023). In addition, studies have demonstrated that Atlantic salmon in the freshwater phase is mostly PRV‐1 negative (Sommerset et al. 2024). Most cases of infection occur in seawater, either immediately after transport to the sea or within 8 to 9 months post‐sea transfer. Moreover, it cannot be ruled out that the used PRV‐1 strain utilised may not be pathogenic for larval rainbow trout, as a number of strains exhibit varying degrees of virulence (Bjørgen et al. 2019; Løvoll et al. 2012) or replication of PRV‐1 in rainbow trout larvae takes longer than 28 days to reach the peak. The studies conducted by Purcell et al. (2020) demonstrated that infected rainbow trout had a slower viral replication compared to other salmonids, with the peak viral load around nine weeks after infection. Furthermore, it is recognised that PRV‐1 isolates from Norway typically induce a strong innate antiviral response in blood cells when successfully infecting a host cell (Dahle et al. 2015) and that there is a positive correlation between the viral load of PRV‐1 in heart tissue and the expression of *mx1* (Mikalsen et al. 2012; Saint‐Jean and Pérez‐Prieto 2007). In our experimental cultures, we were only able to show conflicting results for this correlation for PRV‐1 and PRV‐3, as no increase in viral load was detected for PRV‐1 or PRV‐3 at 15°C, and the gene expression of *mx1* did not increase either, but in RTC‐A in 8°C, there was no increase in viral load, although *mx1* was expressed at slightly higher levels, the scatter of the individual values was quite high. Nevertheless, we were able to confirm the correlation of viral load and expression of *mx1* for VHSV and other viruses in our heart cell cultures. Additionally, it is conceivable that the heart is not the organ that is most adversely affected by the viral load of PRV. For instance, it has been demonstrated that the spleen and head kidney exhibited a higher viral RNA load than the heart (Løvoll et al. 2010). A further potential explanation for the absence of replication in cardiomyocytes may be linked to temperature. It has been demonstrated that rainbow trout are more effective at eliminating PRV‐3 at warmer temperatures (Sørensen et al. 2023) and disease outbreaks frequently occurring during the winter and spring months when water temperatures are lower (Hauge et al. 2017). The host‐pathogen interaction can be influenced by temperature, which can lead to changes in the immune response of the fish or virus replication (Bowden et al. 2007; Páez et al. 2021). A study by Sørensen et al. (2023) explored the possible effects of water temperature on PRV‐3 infection in rainbow trout and demonstrated that the expression of antiviral genes like *ifit5*, *isg15*, *mx1* and other genes was increased at all temperatures (5°C, 12°C and 15°C) considered. This finding aligns with those of other studies on PRV, which showed also that infection with PRV triggers an antiviral response (Dahle et al. 2015; Wessel et al. 2017).

For the reasons mentioned above, an attempt was also made to infect cardiomyocyte cultures from one‐year‐old rainbow trout (RTC‐A) as representatives of the next developmental stage with PRV‐1 and incubate them at 8°C. Furthermore, the same heart cell cultures were infected with VHSV and incubated at the same temperature. VHSV is a virus that is known to infect multiple organs like the heart, kidney and spleen (Gary et al. 1998; Maj‐Paluch et al. 2022), induces a strong antiviral response (Pham et al. 2013; Simón et al. 2023) and is capable of infecting and replicating in cell cultures as previously shown by Bauer et al. (2023) and Quillet et al. (2001). A previous study has demonstrated that during VHSV infection and replication in the RTG‐2 cell line, genes such as *vig1* and *mx1* as well as *ifn1* are markedly upregulated (Adamek et al. 2021). When using the heart cell cultures in our study we could confirm that both the viral load in the cells and in the cell medium increased significantly and antiviral genes were significantly upregulated by the infection. Thus, gene expression is dependent on viral load and viral replication was confirmed at both levels. Therefore, VHSV serves as a positive control for us, confirming that viruses are capable of replicating in the beating cardiomyocyte cultures. Previous findings on PRV‐1 showed that PRV‐1 first replicates in red blood cells (Wessel et al. 2015) and secondly thereafter in cardiomyocytes (Dhamotharan et al. 2020; Polinski et al. 2020). We wanted to test these findings in our heart cell cultures, as the expanded characteristic of contraction enabled the creation of a cell culture that more closely resembles the heart. This represents a new possibility for in vitro PRV cultivation. However, no increase in viral RNA load was observed in the cells nor in the medium for PRV‐1 at 8°C. In contrast to the 15°C infection experiment, however, an upregulation of the antiviral genes *isg15*, *mx1* and *sacs* was observed. Nevertheless, the degree of upregulation is relatively modest, and there is considerable variability in gene expression within the PRV‐1 groups. The slight increase in the expression of antiviral genes can also be attributed to this phenomenon, as the presence of PRV‐1 both in the cells and in the cell medium leads to the activation of antiviral defence mechanisms. However, due to the relatively low viral load, there is no significant regulation of gene expression raised to eliminate the viruses.

As a final aspect of our study, a comparison was conducted between the new beating cardiomyocyte cell cultures (RTC‐A) and the established cell cultures RTG‐2 (Wolf and Quimby 1962) and new established permanent cell line from heart fibroblasts RTH‐F to evaluate their respective performances during infection with additional fish viruses. Using the cardiomyocytes (RTC‐A) and fibroblasts from the heart (RTH‐F) allowed us to better characterise a cardiotropic character of the viruses. The results obtained from the analysis of the CSV in our cell cultures are inconclusive. In RTC‐A, there is an increase in the viral load, whereas in RTG‐2 and RTH‐F, no significant change is observed. However, at the gene expression level, there is an increase in both *mx1* and *vig1* transcription in all infected cell cultures. The observed differences in viral load may be attributed to the divergent ways in which the cell lines respond to the virus's dsRNA (Jensen, Larsen, and Robertsen 2002). It thus appears that RTC‐A may represent a novel cell culture for CSV replication. Moreover, it is established that RTG‐2 produces only a limited quantity of the virus (Winton et al. 1981). In the RTG‐2 model, the observed effect of CSV was primarily indirect and dependent on type I IFN induction, which in turn resulted in the upregulation of *mx* and *vig‐1* transcripts (DeWitte‐Orr, Leong, and Bols 2007). As with our findings in CSV, the IPNV results also demonstrate dissimilar outcomes across the three cell lines. While the virus is evidently replicating in RTC‐A after 1 dpi, it has already returned to baseline by 5 dpi. Conversely, an increase in viral load is exclusively observed in the other heart cell culture, RTH‐F, after five dpi. The expression of the antiviral genes correlates with the viral load in the cell lines. The results are therefore in accordance with the findings of other studies which have demonstrated that IPNV frequently replicates in fish cell lines (Løkka et al. 2023; Sadasiv 1995). Perhaps more unexpectedly, no visible replication occurred in RTG‐2. It is known that IPNV can trigger the regulation of gene expression levels without replicating inside the cells (Nombela et al. 2017). Conversely, the replication of IPNV in RTG‐2 remains a source of perplexity, suggesting that RTG‐2 may not be the optimal cell line for this purpose (Kelly, Souter, and Miller 1978; Lorenzen, Carstensen, and Olesen 1999).

The results for the other heart‐related viruses were found to be inconsistent. We wanted to ascertain whether PMCV is capable of replication in our beating heart cell cultures derived from rainbow trout, as well as in the other two rainbow trout cell lines, even though PMCV is a virus that has only been observed to infect Atlantic salmon. It is known to cause cardiomyopathy syndrome (CMS), which is diagnosed by histopathology. PMCV is characterised by severe inflammation and degeneration of the spongious part of the ventricle and atrium (Haugland et al. 2011). The disease develops at a relatively slow pace, with the CMS phenomenon typically manifesting itself approximately six weeks after the initial viral infection (Fritsvold et al. 2009; Sun et al. 2024a). PMCV was not detected in any of the three cultures. Given the slow development of the disease and the necessity to ascertain whether PMCV infects rainbow trout, further research is required in the form of, for example, longer observation periods following the infection of cell cultures. Nevertheless, our results offer initial insights into the replication of PMCV in rainbow trout. In contrast to PMCV, SAV‐3 replication occurred in the heart‐related cultures RTC‐A and RTH‐F, both in terms of increase in viral load and the induction of antiviral response, but not in the cell line RTG‐2 that shows fibroblast‐like morphology and was derived from ovary and testis. Our findings for SAV‐3 are mostly consistent with those of other studies that have demonstrated the susceptibility of Atlantic salmon heart explant cultures to SAV‐3 (Noguera et al. 2017). Rainbow trout was also consistently confirmed to be susceptible to SAV‐3 in vivo and in vitro (Deperasińska, Schulz, and Siwicki 2018; Løkka et al. 2023; Taksdal et al. 2007). In previous studies, following the infection of cell lines derived from rainbow trout (RTgutCC) or Atlantic salmon heart explant cultures with SAV‐3, a strong antiviral response was observed, indicating that the expression of *mx1* may serve as a potential marker to confirm viral infection and replication of SAV‐3 also in our cell cultures (Løkka et al. 2023; Noguera et al. 2017). However, *mx1* was merely upregulated in RTC‐A and RTH‐F. Compared to RTG‐2, which showed no replication or antiviral response, especially RTC‐A appears to be a promising cell culture for the replication of SAV‐3. The TiLV results demonstrate a slight replication in RTG‐2 and RTH‐F, but not in RTC‐A. However, at the gene expression level, there is an increase in the expression of *vig1* and *mx1* in all cell lines. Our findings are in accordance with previous research indicating that TiLV can replicate in RTG‐2 at 15°C. However, this study demonstrated that higher temperatures are more suitable for virus replication (Adamek et al. 2023).

In conclusion, the objective of this study was to test novel cell cultures of rainbow trout cardiomyocytes from different developmental stages to ascertain the potential for PRV‐1 and PRV‐3 replication. Furthermore, the susceptibility of this cell culture to other cardiac and fish viruses was tested, and it was compared to the new cell line RTH‐F and established cell line RTG‐2. Replication of PRVs was not observed in our RTC cell line at 8°C or 15°C. No indicators of viral replication for PRVs like the viral load in the cells or in the cell medium or the expression of the antiviral genes analysed showed any significant changes during the infection experiments. However, the increase in viral replication indicators confirmed the replication of CSV, IPNV and VHSV. Therefore, this novel cell culture contributes to the advancement of in vitro virus research and provides a valuable new tool in this field.

## Author Contributions

**Torben Krebs:** conceptualization, methodology, data curation, investigation, visualization, resources, writing – original draft, writing – review and editing. **Julia Bauer:** investigation, conceptualization, methodology, writing – review and editing. **Sarah Graff:** methodology, resources, writing – review and editing. **Lukas Teich:** investigation, writing – review and editing. **Markus Sterneberg:** investigation, writing – review and editing. **Marina Gebert:** conceptualization, resources, project administration, writing – review and editing. **Henrike Seibel:** conceptualization, writing – review and editing, resources, project administration. **Bettina Seeger:** methodology, formal analysis, writing – review and editing. **John Hellmann:** resources, writing – review and editing. **Øystein Wessel:** resources, writing – review and editing. **Espen Rimstad:** resources, writing – review and editing. **Win Surachetpong:** resources, writing – review and editing. **Dieter Steinhagen:** writing – original draft, supervision, formal analysis, writing – review and editing. **Verena Jung‐Schroers:** supervision, writing – review and editing. **Mikolaj Adamek:** writing – review and editing, writing – original draft, funding acquisition, validation, visualization, project administration, supervision, data curation, methodology, resources, conceptualization.

## Ethics Statement

The research was conducted on non‐feeding larvae and tissues collected from fish post‐mortem in accordance with local and international animal experimentation legislation (Article 4, TSchG Killing for scientific purposes, Annunciation number: TiHo‐T‐2022‐3).

## Conflicts of Interest

The authors declare no conflicts of interest.

## Supporting information


**Table S1** Primer sequences used in this work. All primer sequences were used in real‐time qPCR gene mRNA expression analysis.

## Data Availability

Data are available within the article or its supplementary materials.
