# Supplementary material for: Beating Cardiac Cell Cultures From Different Developmental Stages of Rainbow Trout as a Novel Approach for Replication of Cardiac Fish Viruses
Source: J Fish Dis. 2025 Jan 17;48(5):e14080. doi: 10.1111/jfd.14080 (PMC11976189; doi:10.1111/jfd.14080)
Supplement: Supplementary file 1 — Table S1 Primer sequences used in this work. All primer sequences were used in real‐time qPCR gene mRNA expression analysis. [file JFD-48-e14080-s001.docx]

**Supplementary data:**

**Table S1:** Primer sequences used in this work. All primer sequences were used in real-time qPCR gene mRNA expression analysis.

| **Target** | **Primer Name** | **Primer/Probe sequence** | **References/Accession number** |
| --- | --- | --- | --- |
| **Viral genes** |  |  |  |
| CSV | CSV_qF1 | CATGAACGTCCCCTCATCC | (Rakus et al., 2019) |
|  | CSV_qR1 | ATATAGACGCGCCAGGTGTT |  |
| IPNV | IPN_WB_qF | CCGCAACTTACTTGAGATCCATTATGC | (Williams et al., 1999) |
|  | IPN_WB_qR | CGTCTGGTTCAGATTCCACCTGTAGTG |  |
| PMCV | PMCV_ORF1_qF1 | GAGGRTTYTGGGCGCAAC | This communication |
|  | PMCV_ORF1_qR1 | YGGTCTCACYCCTCTCTCC |  |
|  | PMCV_ORF1_qProbe1 | FAM-ACTAGGAGGATTGGCAGGGT-BHQ1 |  |
| PRV-1 | PRV_Sybr_qF | AATTGAAGACCTCCCCATCC | (Adamek et al., 2019) |
|  | PRV_Sybr_qR | GGTGAAATCATCGCCAACTC |  |
| PRV-3 | PRV_Om_Sybr_qF2 | CGGGCATAGGATTGGTCTGT | (Olsen et al., 2015) |
|  | PRV_Om_Sybr_qR2 | GGTAGGCGTCGGTGTTTG |  |
| SAV-3 | SAV_TE2_qF1 | TTACGACGGTGCTAATCTCTAC | (Shi et al., 2017) |
|  | SAV_TE2_qR1 | ACCAAGTAACGGAGAAGTTCAC |  |
| TiLV | TiLV_93F | AGCCTGCCACACAGAAG | (Waiyamitra et al., 2018) |
|  | TiLV_93R | CTGCTTGAGTTGTGCTTCT |  |
|  | TiLV_93Probe | FAM-CTCTACCAGCTAGTGCCCCA-BHQ |  |
| VHSV | VHSV_N_qF1 | GAATCCGTGCAGCTTTTTCAGG | (Kim et al., 2014) |
|  | VHSV_N_qR1 | CAAGTGCATCCACGATCACCTTC |  |
| **Salmonid genes** |  |  |  |
| *ef1α* | Onmy_ef1α_qF1 | TGGGCTGGTTCAAGGGATGG | (Dietrich et al., 2015) |
|  | Onmy_ef1α_qR1 | CTGGAGGGGCAGACGAAGG |  |
| *ifng2* | Onmy_ifng2_qF1 | CCTGTTTTCCCCAAGGACAC | (Purcell et al., 2009) |
|  | Onmy_ifng2_qR1 | CCAGCCTCTCCCTCACTTC |  |
| *il-6l* | Onmy_il-6l_qF1 | GGCTGAATACCCACAATCCA | XM_021572949 |
|  | Onmy_il-6l_qR1 | TAGACACCTCACCCAGCACA |  |
| *isg15* | Onmy_isg15_qF1 | GGTTCAGCCACACACCACTC | (O'Farrell et al., 2002) |
|  | Onmy_isg15_qR1 | ACAGCCTCTGCCTTTCCA |  |
| *mx1* | Onmy_mx1_qF1 | GGTTGTGCCATGCAACGTT | (Park et al., 2011) |
|  | Onmy_mx1_qR1 | GGCTTGGTCAGGATGCCTAAT |  |
| *sacs* | Onmy_sacs_qF1 | ATCAAATGGCAGGAGGAAGA | XM_021584972 |
|  | Onmy_sacs_qR1 | GCAGCATCAGGAGGTATGTG |  |
| *vig1* | Onmy_vig1_qF1 | ACAAAGTGGCGTTCAAAATC | NM_001124253 |
|  | Onmy_vig1_qR1 | ACTGTTCTCCCCAGCGTTC |  |
| *39SL40* | Sasa_39SL40_qF1 | CCCAGTATGAGGCACCTGAAGG | (Deeg et al., 2022) |
|  | Sasa_39SL40_qR1 | GTTAATGCTGCCACCCTCTCAC |  |

Adamek, M., Hellmann, J., Flamm, A., Teitge, F., Vendramin, N., Fey, D., Riße, K., Blakey, F., Rimstad, E., Steinhagen, D., 2019. Detection of piscine orthoreoviruses (PRV-1 and PRV-3) in Atlantic salmon and rainbow trout farmed in Germany. Transboundary and Emerging Diseases 66, 14-21.

Deeg, C.M., Kanzeparova, A.N., Somov, A.A., Esenkulova, S., Di Cicco, E., Kaukinen, K.H., Tabata, A., Ming, T.J., Li, S., Mordecai, G., Schulze, A., Miller, K.M., 2022. Way out there: pathogens, health, and condition of overwintering salmon in the Gulf of Alaska. FACETS 7, 247-285.

Dietrich, M.A., Nynca, J., Adamek, M., Steinhagen, D., Karol, H., Ciereszko, A., 2015. Expression of apolipoprotein A-I and A-II in rainbow trout reproductive tract and their possible role in antibacterial defence. Fish Shellfish Immunol 45, 750-756.

Kim, J.O., Kim, W.S., Kim, S.W., Han, H.J., Kim, J.W., Park, M.A., Oh, M.J., 2014. Development and application of quantitative detection method for viral hemorrhagic septicemia virus (VHSV) genogroup IVa. Viruses 6, 2204-2213.

O'Farrell, C., Vaghefi, N., Cantonnet, M., Buteau, B., Boudinot, P., Benmansour, A., 2002. Survey of transcript expression in rainbow trout leukocytes reveals a major contribution of interferon-responsive genes in the early response to a rhabdovirus infection. J Virol 76, 8040-8049.

Olsen, A.B., Hjortaas, M., Tengs, T., Hellberg, H., Johansen, R., 2015. First Description of a New Disease in Rainbow Trout (Oncorhynchus mykiss (Walbaum)) Similar to Heart and Skeletal Muscle Inflammation (HSMI) and Detection of a Gene Sequence Related to Piscine Orthoreovirus (PRV). PLoS One 10, e0131638.

Park, J.W., Moon, C.H., Harmache, A., Wargo, A.R., Purcell, M.K., Bremont, M., Kurath, G., 2011. Restricted growth of U-type infectious haematopoietic necrosis virus (IHNV) in rainbow trout cells may be linked to casein kinase II activity. J Fish Dis 34, 115-129.

Purcell, M.K., Laing, K.J., Woodson, J.C., Thorgaard, G.H., Hansen, J.D., 2009. Characterization of the interferon genes in homozygous rainbow trout reveals two novel genes, alternate splicing and differential regulation of duplicated genes. Fish Shellfish Immunol 26, 293-304.

Rakus, K., Adamek, M., Mojżesz, M., Podlasz, P., Chmielewska-Krzesińska, M., Duk, K., Kasica-Jarosz, N., Kłak, K., Rakers, S., Way, K., Steinhagen, D., Chadzińska, M., 2019. Evaluation of zebrafish (Danio rerio) as an animal model for the viral infections of fish. Journal of Fish Diseases 42, 923-934.

Shi, W., Song, A., Gao, S., Wang, Y., Tang, L., Xu, Y., Ren, T., Li, Y., Liu, M., 2017. Rapid and sensitive detection of salmonid alphavirus using TaqMan real-time PCR. Molecular and Cellular Probes 34, 13-20.

Waiyamitra, P., Tattiyapong, P., Sirikanchana, K., Mongkolsuk, S., Nicholson, P., Surachetpong, W., 2018. A TaqMan RT-qPCR assay for tilapia lake virus (TiLV) detection in tilapia. Aquaculture 497, 184-188.

Williams, K., Blake, S., Sweeney, A., Singer, J.T., Nicholson, B.L., 1999. Multiplex reverse transcriptase PCR assay for simultaneous detection of three fish viruses. J Clin Microbiol 37, 4139-4141.
